# Prevalence and correlates of anemia among HIV infected patients on highly active anti-retroviral therapy at Zewditu Memorial Hospital, Ethiopia

**DOI:** 10.1186/s12878-015-0024-6

**Published:** 2015-04-30

**Authors:** Muluken Assefa, Woldaregay Erku Abegaz, Aster Shewamare, Girmay Medhin, Mulugeta Belay

**Affiliations:** Mizan Aman Health Science College, Southern Nations, Nationalities and Peoples’ Regional Health Bureau, Mizan-Aman, Ethiopia; Aklilu Lemma Institute of Pathobiology, Addis Ababa University, Addis Ababa, Ethiopia; Zewditu Memorial Hospital ART Clinic, Addis Ababa, Ethiopia

**Keywords:** HIV, HAART, Anemia, Ethiopia

## Abstract

**Background:**

Ethiopia is one of the most seriously HIV affected countries in Sub-Saharan Africa. Anemia is a known predictor of disease progression and death among HIV infected patients. In this study, we investigated the magnitude and correlates of anemia among HIV infected patients receiving HAART at a referral hospital in Ethiopia.

**Methods:**

A retrospective cohort study was conducted from November 2011 to February 2012 in Zewditu Memorial Hospital, Addis Ababa, Ethiopia. Records of 1061 patients on HAART were selected using simple random sampling technique. Socio-demographic and clinical characteristics of the study patients were collected using standardized data extraction instrument. Data were analyzed using STATA version 11.0. Odds ratios with 95% confidence intervals were used to quantify the strength of association between anemia and its potential predictors.

**Results:**

The prevalence of anemia at baseline was 42.9%. However, the prevalence significantly decreased to 20.9% at 6 months (p < 0.001) and to 14.3% at 12 months (p = 0.001) after HAART initiation. At baseline, male sex (AOR = 1.55; 95% CI: 1.18-2.03), clinical stage III/IV (AOR = 2.03; 95% CI: 1.45-2.83) and TB co-infection (AOR = 1.52; 95% CI: 1.08-2.13) were independently associated with the odds of being anemic. After 6 months of HAART, male sex (AOR = 1.59; 95% CI: 1.13-2.23), baseline anemia (AOR = 2.38; 95% CI: 1.71-3.33) and TDF-based HAART (AOR = 2.87; 95% CI: 1.80-4.60) were independently associated with the odds of being anemic. Besides, anemia was independently associated with older age at 6 months. After 12 months of HAART, baseline anemia (AOR = 2.01; 95% CI: 1.36-2.97), age group 25–34 years (AOR = 5.92; 95% CI: 1.39-25.15), age group 45–54 years (AOR = 4.78; 95% CI: 1.07-21.36), CD4 count below 200 cells/mm^3^ (AOR = 2.15; 95% CI: 1.21-3.82) and 200–350 cells/mm^3^ (AOR = 1.91; 95% CI: 1.13-3.25) were independently associated with the odds of being anemic.

**Conclusions:**

Although a remarkable reduction in the prevalence of anemia was observed following initiation of HAART, a significant proportion of HIV patients remained anemic after 12 months of HAART suggesting the need for routine screening and proper treatment of anemia to mitigate its adverse effects.

## Background

Complications of human immunodeficiency virus (HIV) infection include hematological abnormalities manifested by pancytopenia, anemia being the leading abnormality [1-3]. However, the prevalence of anemia in HIV patients varies considerably, ranging from 1.3% to 95% [4]. Several factors including stage of HIV, age and sex are said to account for the variations in HIV prevalence [4]. The causes of anemia have been reported to be multifactorial. Direct effects of HIV and its viral proteins as well as immune dysregulations during HIV infection were found to be responsible for bone marrow suppression [5,6]. Moreover, opportunistic infections of the bone marrow with pathogens such as *Mycobacterium avium complex*, *Parvovirus B-19*, Cytomegaloviruses, *Cryptococcus neoformans* and *Histoplasma capsulatum* were reported to cause abnormalities in blood cell counts [6,7]. Drugs used to treat HIV infection and its complications are also known to cause bone marrow suppression [7,8]. It is widely known that AZT alone and AZT based highly active antiretroviral treatment (HAART) regimen is associated with significant reduction of hemoglobin (Hgb) level [7,9,10].

Anemia is associated with impaired physical functioning, psychological distress and poor quality of life [7]. Besides, independent of CD4 and viral load counts, anemia has been reported to predict HIV progression to acquired immune deficiency syndrome (AIDS) with poor survival [4,7,11,12]; on the other hand, treatment of anemia was observed to be associated with reversal of increased risk of death [13]. In addition, anemia was reported to be strongly and consistently associated with HIV disease progression and death despite HAART initiation [8], suggesting the need for routine screening and treatment of anemia in HIV patients on HAART. Prevalence of anemia among Ethiopian men and women aged 15–49 years was documented to be 11% and 17%, respectively [14]. Regional differences were remarkable and the prevalence of anemia in men and women aged 15–49 years in Addis Ababa were 3% and 9%, respectively [14]. In addition, recent study reported a considerably higher prevalence of anemia among HIV-infected antiretroviral-naïve adult Ethiopians (38% for men and 62% for women) [15]. Ethiopians’ normal immunohematological profile is known to be lower than the corresponding values in Caucasians [16]. Besides, AZT-based HAART is one of the first line regimens recommended for treating HIV infected adults [17]. However, the magnitude of anemia and its risk factors among HIV patients on HAART are not well document in Ethiopia. Thus, this study investigated the prevalence of anemia and its associated factors among HIV infected patients receiving HAART in a referral hospital in Ethiopia.

## Methods

### Study setting and study population

A retrospective cohort study was conducted by reviewing hospital data of patients who were receiving HAART at the HIV clinic of Zewditu Memorial Hospital, Addis Ababa, Ethiopia from November 2011 to February 2012. The Hospital launched Antiretroviral Therapy (ART) in July 2003 as the first pilot site in the country. Among 15,000 HIV infected adults on care at the Hospital, 9,287 were on ART during data collection. Among those on ART, a total of 1061 HIV infected adults were randomly selected and included in this study. Sample size was estimated using a single population proportion formula, taking p=46% (expected prevalence of anemia at baseline) [1], 3% level of precision (d) with 95% confidence interval. Patients transferred in from other health institutions, patients who started ART in the hospital before March 1, 2005 or after November 30, 2010 and patients who were pregnant at baseline or during the follow up visits were excluded from the study.

### Data collection

The data was extracted using a standardized instrument by three ART nurses. The collected information includes socio-demographics, clinical characteristics, medication use and immunohematological profile of patients at baseline, 6 and 12 months after initiation of HAART. Hemoglobin (Hgb) values, red blood cell count, white blood cell counts with absolute CD4 cells and CD8 cells, and platelets were determined using the hematology analyzer Cell-Dyn 1800 (Abbott Laboratories Diagnostics Division, USA) whereas CD4+ T cells were assayed using the BD FACSCOUNT system (Becton Dickenson and Company, California, USA). To ensure good quality data, training of data collectors, pre- testing of data extraction instrument and continuous supervision of the data collection process was carried out. Data were checked for missed values and outliers during data management.

### Definition of outcome variable and statistical analysis

Data were computerized using Epidata version 3.1 and STATA version 11 (StataCorp LP, college station, TX) was used for data analysis. Anemia was defined based on hematological reference values for adult Ethiopian population [16]. Accordingly, anemia was defined as Hgb concentration less than or equal to 13.9 g/dl for adult males and less than or equal to 12.2 g/dl for adult females. It was further classified into mild (10–12.2 g/dl for females and 10–13.9 g/dl for males), moderate (8–10 g/dl) and severe (<8 g/dl) for both sexes.

The effect of different risk factors on the odds of being anemic were investigated at baseline, at 6 months and at 12 months following initiation of HAART. Odds ratio with 95% confidence interval was used to quantify the strength of association between anemia and its potential predictors. Variables that were found significantly associated with the outcome variable (p < 0.05) in the bivariate analysis as well as variables that were reported to be associated with anemia in previous studies and that were clinically relevant for the occurrence of anemia were included in the multivariable logistic regression model to obtain adjusted effects. Since height was not recorded for the majority of the study participants, missing values for body mass index (BMI) were coded as a separate category before including BMI in multivariable logistic regression so that data from all study participants can be used.

### Ethical consideration

Study protocol was approved by the Institutional Review Board of Aklilu Lemma Institute of Pathobiology, Addis Ababa University and Addis Ababa City Administration Health Bureau. Identifiers of the study participants were not collected to maintain confidentiality.

## Results

### Baseline characteristics of patients

Demographic and clinical characteristics of the study patients at treatment initiation are presented in Table 1 and Table 2, respectively. Of the total 1061 study patients, 60% were females, 76.0% were in the age range of 25–49 years and the overall age range was 17–88 years. Majority (62.6%) of the patients were classified as WHO clinical stage III/IV and 79.4% had a CD4 cell count of less than 200 cells/mm^3^. The mean Hgb value was 12.9 (sd = 2.4 g/dl) while the mean CD4 cell count was 139.8 (sd = 78.8) cells/mm^3^. Five hundred and forty-four (52.0%) of the study patients were co-infected with different types of opportunistic infections.

**Table 1:** **Baseline socio-demographic characteristics of HIV infected patients on HAART at Zewditu Memorial Hospital, Addis Ababa, Ethiopia, 2012**

**Variable**	**Frequency**	**Percent**
**Gender (n = 1061)**		
Female	632	59.6
Male	429	40.4
**Age (in years) (n = 1059)**		
15-24	64	6.0
25-34	420	39.7
35-44	385	36.4
45-54	151	14.2
55+	39	3.7
**Religion (n = 1013)**		
Muslim	64	6.3
Orthodox	827	81.6
Protestant	116	11.5
Catholic	5	0.5
Other	1	0.1
**Educational status (n = 1008)**		
No education	97	9.6
Primary	332	32.9
Secondary	425	42.2
Tertiary	154	15.3
**Marital status (n = 1031)**		
Never married	223	21.6
Married	513	49.8
Separated	27	2.6
Divorced	110	10.7
Widowed	158	15.3
**Employment status (n = 629)**		
No	262	41.7
Yes	367	58.3

**Table 2:** **Baseline clinical characteristics of HIV infected patients on HAART at Zewditu Memorial Hospital, Addis Ababa, Ethiopia, 2012**

**Variable**	**Frequency**	**Percent**
**WHO clinical stage (n = 1059)**		
Stage I /II	396	37.4
Stage III /IV	663	62.6
**CD4 cell count (Cells/mm3) (n = 1058)**		
≥350	14	1.3
200-350	204	19.3
0-199	840	79.4
*BMI (Kg/m2) (n = 243)		
<18.5	54	22.2
18.5-24.9	158	65.0
≥25.0	31	12.8
**Opportunistic infections (n = 1060)**		
No OI	508	47.9
Candidiasis	251	23.7
Herpes zoster	259	24.4
Tuberculosis	228	21.5
Fever	49	4.6
Diarrhea	63	5.9
Bacterial pneumonia	33	3.1
Other OIs	30	2.8

OI: Opportunistic Infection; *BMI: Body mass index.

### Anemia before HAART initiation

A total of 455 (42.9%) patients were anemic at baseline of which 360 (34.0%) had mild, 71 (6.7%) had moderate and 24 (2.3%) had severe anemia. The overall prevalence of anemia was higher in males (50.4%) than in females (37.8%) (p < 0.001). Significantly higher proportion of females had moderate and severe forms of anemia compared to males: moderate anemia was 7.9% among females and 4.9% among males (p < 0.001) and severe anemia (2.9% among females and 1.4% among males (p < 0.001).

Different factors were observed to be associated with being anemic before HAART initiation (Table 3). In univariable analysis, increased risk of anemia at baseline was associated with male sex (OR = 1.67; 95% CI: 1.30-2.14), age greater than 55 years (OR = 2.40; 95% CI: 1.06-5.41), clinical stage III/IV (OR = 2.35; 95% CI: 1.81-3.06), BMI <18.5 (OR = 2.32; 95% CI: 1.24-4.36) and the presence of opportunistic infections such as TB (OR =2.07; 95% CI: 1.54-2.80) and candidiasis (OR = 1.44; 95% CI: 1.08-1.91). CD4 count <200 cells/mm^3^ and 200-350 cells/mm^3^ are associated with decreased odds of being anemic. In multivariable logistic regression analysis, male sex, clinical stage III/IV and TB co-infection were independently associated with increased odds of being anemic whereas CD4 count <200 cells/mm^3^ and 200-350 cells/mm^3^ were independently associated with reduced odds of being anemic in the multivariable analysis.

**Table 3:** **Predictors of anemia in HIV Infected patients at before HAART initiation at Zewditu Memorial, Addis Ababa, Ethiopia 2012**

**Variable**	**Crude OR (95% CI)**	**AOR (95% CI)**
**Gender**		
Female	1.00	1.00
Male	1.67 (1.30-2.14)	1.55 (1.18-2.03)
**Age (in years)**		
15-24	1.00	1.00
25-34	0.99 (0.58-1.71)	0.99 (0.56-1.77)
35-44	1.46 (0.85-2.52)	1.46 (0.82-2.60)
45-54	1.40 (0.77-2.55)	1.21 (0.64-2.29)
55+	2.40 (1.06-5.41)	2.06 (0.88-4.82)
**WHO clinical stage**		
Stage I/II	1.00	1.00
Stage III/IV	2.35 (1.81-3.06)	2.03 (1.45-2.83)
**CD4 cell count (cells/mm** ^**3**^ **)**		
≥350	1.00	1.00
200-350	0.13 (0.03-0.47)	0.16 (0.04-0.61)
0-199	0.22 (0.06-0.81)	0.25 (0.07-0.93)
**BMI**		
Normal	1.00	1.00
Underweight	2.32 (1.24-4.36)	1.83 (0.92-3.65)
Overweight	0.95 (0.42-2.12)	1.14 (0.49-2.65)
**OI**		
Candidiasis	1.44 (1.08-1.91)	1.09 (0.78-1.52)
Herpes zoster	0.86 (0.65-1.14)	0.86 (0.64-1.17)
TB	2.07 (1.54-2.80)	1.52 (1.08-2.13)
Diarrhea	0.87 (0.52-1.46)	0.72 (0.40-1.32)
Fever	1.19 (0.67-2.11)	1.00 (0.52-1.96)
Pneumonia	1.84 (0.91-3.71)	1.94 (0.90-4.18)
Toxoplasmosis	1.34 (0.50-3.59)	1.03 (0.36-2.93)

OR: Odds ratio; AOR: Adjusted odds ratio; BMI: Body mass index; OI: opportunistic infections.

### Anemia after HAART initiation

A total of 980 (92.4%) of the study participants had Hgb measurements at 6 months of HAART initiation. Of these, 970 (99%) had Hgb measurements at baseline. And of the 896 (84.4%) patients who had Hgb measurements at 12 month, 888 (99.1%) had Hgb measurements both at baseline and at 6 months of HAART treatment. Patients with missing Hgb measurements at 6 month (81 patients) were more likely to be those in the age group of 25-34 years, those having a CD4 cell counts of ≥350 cells/mm3, those who were not on co-trimoxazole prophylactic treatment and those who were receiving d4T based HAART regimen. However, there was no statistically significant difference in the sex, BMI values, fluconazole treatment status, TB treatment status and anti-microbial treatment status between patients included and excluded from the study of anemia at the 6 month. During the study period, changes were made to the regimen of 85 patients. Of the 60 patients for whom drug regimen was changed in the first 6 months, 25(41.7%) were those who were anemic at baseline. Treatment regimen was changed for 40 of the patients who were on AZT based regimen (15 (37.5%) were anemic at baseline), for 16 of those on d4T- based regimen (9(56.3%) were anemic at baseline) and for 4 of those on TDF- based regimen (1(25.0%) were anemic at the baseline). After 6 months, 25 patients have changed their treatment regimens of whom 18 (72%) were patients on d4T- based regimen and 7 (28%) were those on AZT- based regimen. The mean (sd) Hgb values of the study patients at 6 and 12 months were 14.0 (2.3) g/dl and 14.4 (1.9) g/dl, respectively.

**Figure 1:**
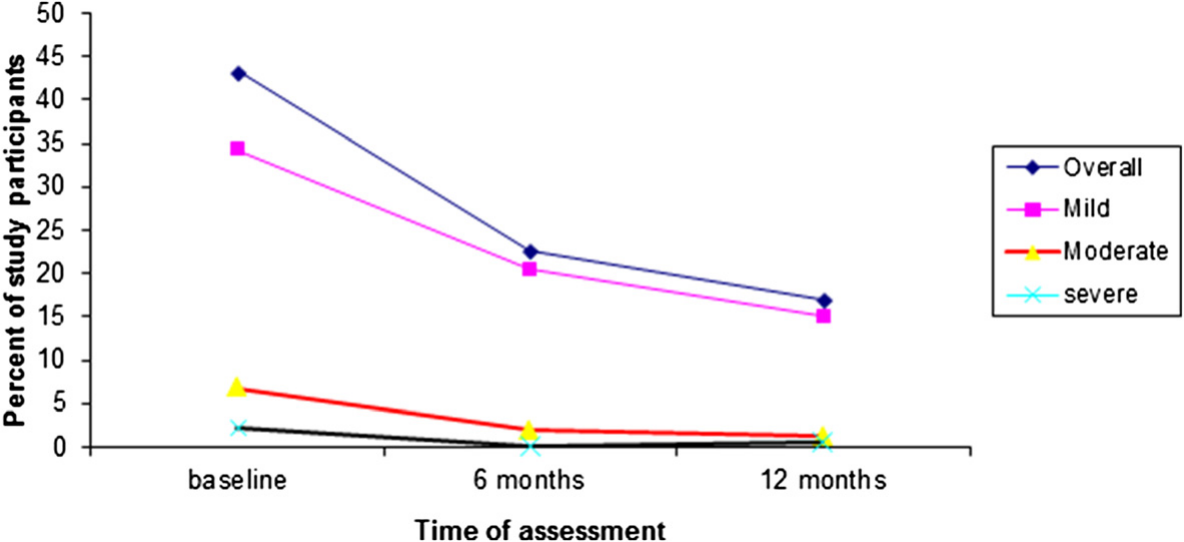
Changes in the prevalence of anemia according to severity grades after HIV infected patients started HAART in Zewditu Memorial Hospital, Addis Ababa, Ethiopia, 2012.

The overall prevalence of anemia significantly decreased after HAART initiation. The prevalence declined from 42.9% at baseline to 20.9% at 6 months (p < 0.001) and to 14.3% at 12 months (p = 0.001). There was also a consistent decrease in the prevalence of mild, moderate and severe anemia after treatment with HAART (Figure 1). Similar to the baseline level, anemia remained to be more prevalent among males compared to females after 6 months of treatment with HAART. At 6 months following initiation of HAART, 25.4% of males and 17.9% of females were anemic. At the same time, more males than females had mild anemia (24.2% versus 15.4%), moderate anemia (2.4% versus 1.2%) and severe anemia (0.2% versus 0.0%) (p = 0.002). However, there was no statistically significant difference in the prevalence of anemia between males and females at 12 months of HAART.

Risk factors for anemia after initiation of HAART are shown in Table 4. At 6 months of HAART, male sex (AOR = 1.59; 95% CI: 1.13-2.23), anemia at baseline (AOR = 2.38; 95% CI: 1.71-3.33), and TDF- based HAART regimen (AOR = 2.87; 95% CI: 1.80-4.60) were independent predictors of anemia. Besides, at 6 months, older age was independently associated with increased odds of being anemic. At 12 months, anemia at baseline (AOR = 2.01; 95% CI: 1.36-2.97), being in the age range of 25–34 years (AOR = 5.92; 95% CI: 1.39-25.15), being in the age range of 45–54 years (AOR = 4.78; 95% CI: 1.07-21.36), CD4 count below 200 cells/mm^3^ (AOR = 1.91; 95% CI: 1.13-3.25) and 200–350 cells/mm^3^ (AOR = 2.15; 95% CI: 1.21-3.82) were independently associated with increased odds of being anemic.

**Table 4:** **Predictors of anemia in HIV Infected patients at 6 and 12 months of HAART initiation at Zewditu Memorial Hospital, Addis Ababa, Ethiopia, 2012**

**Variable**	**6 months**		**12 months**	
	**Crude OR (95% CI)**	**AOR (95% CI**	**Crude OR (95% CI)**	**AOR (95% CI**
**Gender**				
Female	1.00	1.00	1.00	1.00
Male	1.56 (1.16-2.11)	1.59 (1.13-2.23)	1.19 (0.84-1.68)	1.01 (0.68-1.50)
**Age (in years)**				
15-24	1.00	1.00	1.00	1.00
25-34	2.99 (1.17-7.69)	3.50 (1.31-9.33)	4.14 (1.26-13.55)	5.92 (1.39-25.15)
35-44	3.14 (1.22-8.09)	3.09 (1.16-8.22)	2.76 (0.83-9.15)	3.27 (0.76-14.11)
45-54	4.40 (1.65-11.73)	4.32 (1.56-11.99)	4.23 (1.23-14.52)	4.78 (1.07-21.36)
55+	4.07 (1.27-13.00)	3.32 (0.99-11.16)	2.99 (0.67-13.29)	2.90 (0.48-17.54)
**CD4 count (cells/mm** ^**3**^ **)**				
≥350	1.00	1.00	1.00	1.00
200-350	1.11 (0.72-1.75)	1.10 (0.69-1.75)	2.07 (1.24-3.46)	1.91 (1.13-3.25)
0-199	1.29 (0.82-2.02)	1.23 (0.76-1.99)	2.31 (1.34-3.99)	2.15 (1.21-3.82)
**Baseline anemia**				
Not anemic	1.00	1.00	1.00	1.00
Anemic	2.64 (1.95-3.58)	2.38 (1.71-3.33)	1.74 (1.23-2.45)	2.01(1.36-2.97)
**Medication history**				
Prophylactic				
Co-trimoxazole	0.81 (0.53-1.25)	0.48 (0.17-1.32)	0.68 (0.40-1.14)	0.81 (0.32-2.11)
Others	0.83 (0.34-2.04)	0.69 (0.27-1.76)	0.28 (0.04-2.14)	0.36 (0.05-2.78)
Non-prophylactic				
Anti- TB drug	1.69 (1.08-2.63)	1.11 (0.68-1.82)	2.08 (0.96-4.53)	2.09 (0.90-4.83)
**HAART regimen**				
d4T- based	1.00	1.00	1.00	1.00
AZT- based	0.88 (0.62-1.25)	1.19 (0.81-1.76)	1.15 (0.78-1.69)	1.47 (0.96-2.26)
TDF- based	1.98 (1.30-3.03)	2.87 (1.80-4.60)	0.49 (0.26-0.94)	0.80 (0.40-1.59)

## Discussion

In this study, we investigated the prevalence of anemia and its associated factors in a large cohort of HIV infected patients taking HAART over 12 months. A large proportion of our study patients had anemia at baseline and subsequent follow-up time points. The prevalence of anemia before initiation of HAART in this study was comparable with the prevalence reported in other studies [1,18,19]. However, it was much higher compared to the prevalence reported by Gedefaw et al. from Ethiopia [20], Firnhaber et al. [21] and Mata-Marín et al. [22] elsewhere, who reported a prevalence of 29.9%, 12.0% and 20.0%, respectively. The reasons for the observed difference might be due to the heterogeneity of study population and differences in the study settings in terms of nutritional status, stages of HIV infection and health interventions including treatment of hematological abnormalities. For example, both Firnhaber et al. [21] and Mata-Marin et al. [22] excluded patients with illnesses including opportunistic infections who could have contributed to higher anemia prevalence. Furthermore, the patients in Firnhaber et al. study were from nine countries with diverse geographic settings. A direct comparison of prevalence of anemia in different studies is difficult because of differences in the study population, presentation of data and definition used for anemia. This study used the definition that was proposed specifically for Ethiopian population to define anemia [16].

Results from this study show a remarkable reduction in the prevalence of anemia after initiation of HAART. In agreement with our observation, in other studies, the prevalence of anemia in HAART naïve was significantly higher compared to HAART experienced patients [1,23]. The reduction in opportunistic infections including TB, candidiasis and others, as well as the reduction of inflammatory cytokines such as tumor necrosis factor (TNF) that are implicated in the suppression of erythropoiesis could be mechanisms that may account for the improvement of anemia after initiation of HAART [24,25]. Despite a significant reduction in the prevalence of anemia at 12 months, close to 15% of the patients had anemia implying the need for routine screening of anemia and subsequent investigation of its causes. In this study, based on the data we have, we were not able to determine the specific reasons why these patients had anemia despite treatment with HAART. After 12 months of HAART, low CD4 count was a predictor of anemia suggesting poor recovery and probably high viral load in those patient with anemia after 12 months of HAART. Data on viral load would have been useful but this test is not routinely done at the study site and we were not able to include in the analysis.

Similar to this study, some studies reported a high prevalence of anemia in males compared to females and attributed it to the fact that lower Hgb level is used to define anemia in women than men [26,27]. However, many previous studies reported a high prevalence of anemia in females compared to male HIV infected patients [8,28]. The proposed reason was the presence of menstrual blood loss and to the drains on iron stores that occur with pregnancy and delivery in women*.* However, none of the above studies described the prevalence of anemia according to severity grades. In our study, moderate and severe forms of anemia were higher in females compared to males both before and after 6 months on HAART. The absence of significant difference in the risk of anemia at 12 months on HAART between male and female patients seen in this study may suggest the role of HAART to improve anemia after 12 months.

We observed an increase in the risk of anemia with age. The hematopoietic stem cell displays increasing erythropoietin (EPO) resistance with increasing age and further aging is associated with increased pro-inflammatory cytokine expression many of which contributing to EPO resistance [29], thereby resulting in anemia.

Different studies reported association between increased incidence of low blood cell counts with progressive immunologic deterioration and advanced disease due to HIV [2,8,20]. It was suggested that increasing viral burden as HIV disease progresses could cause hematological abnormality by increasing cytokine mediated myelosuppression [25]. In this study, low CD4 cell count was associated with anemia at baseline although the reasons for such association are not clear. At baseline, the presence of opportunistic infections such as TB and fungal infections like candidiasis might play a major role in the pathogenesis of anemia than the deterioration in immunity as proposed by Scadden and his group [30]. However, an increasing risk in anemia with decreasing values of CD4 cell count was seen at 12 months; and the observed association between anemia and lower CD4 cell count at 12 month might be related to poor immunological recovery and high viral load rather than to opportunistic infections. Increased risk of anemia in patients who were on anti-TB drugs has been observed in this study and it may be related to the anti-TB drugs. More importantly, TB is a chronic infection known to cause moderate degree of anemia [31] which is reversible on treatment.

The absence of significant association between treatment with AZT and anemia in the current study despite its frequent association with bone marrow suppression has also been previously described [32,33]. The effect of AZT may be modest when taken as HAART than administered as a single dose. However, the association of anemia with TDF-based regimen in this study, despite its low cytotoxicity towards erythroid progenitor cells and its effectiveness in terms of viral suppression, CD4 response and reduced adverse events compared to AZT and d4T [34], is largely unexplained and needs further study.

This study is based on a large cohort of HIV patients on HAART. However, data were collected for routine clinical care, and hence, other important variables, notably viral load are lacking. Moreover, the majority of patients had no data on BMI. Nevertheless, this study highlights the burden of anemia among HIV patients before and after HAART.

## Conclusions

In conclusion, anemia is a common manifestation of HIV infection both before and after initiation of HAART in the study setting. The treatment of HIV infected patients with HAART has resulted in a remarkable reduction in the prevalence of anemia after 12 months. However, a substantial proportion of patients had anemia after 12 months of HAART, suggesting the need for routine screening of anemia, investigating its causes and instituting appropriate treatment to mitigate the adverse effects of anemia. Considering the high burden even after a prolonged HAART, there is a need for developing a comprehensive screening, treatment and monitoring protocol for anemia.

## Abbreviations

AIDS: Acquired immune deficiency syndrome
OR: Odds ratio
AOR: Adjusted odds ratio
ART: Anti-retroviral therapy
AZT: Zidovudine
CD4: Cluster of differentiation 4
d4T: Stavudine
HAART: Highly active antiretroviral treatment
Hgb: Hemoglobin
HIV: Human immunodeficiency virus
TB: Tuberculosis
TDF: Tenofovir disoproxil fumarate
